# Adapting the Environmental Scan for Gerontology Graduate Students’ Career Exploration

**DOI:** 10.1093/geroni/igab046.2806

**Published:** 2021-12-17

**Authors:** Nasreen Sadeq, Brianne Stanback

**Affiliations:** University of South Florida, Tampa, Florida, United States

## Abstract

Environmental scanning is a process that provides organizations with information about their internal and external strengths, challenges, and opportunities. Although traditionally used in business and strategic planning, environmental scanning is now being utilized in health care to evaluate currently available programs and services, identify gaps in patient care or research, and make educational, organizational, and policy recommendations. The current study explores adapting the environmental scan for students enrolled in a gerontology graduate program as a tool to facilitate career exploration. Students learned about environmental scanning and were instructed to perform an environmental scan on an important issue, program, or service relevant to their overall career goals. Because students completed their environmental scan while enrolled in an Alzheimer’s Disease Management course, they were encouraged to factor in the increasing prevalence of Alzheimer’s disease into their project. Students’ environmental scan projects spanned several timely topics, including an evaluation of the services currently offered in assisted living facilities from the perspective of a geriatric social worker, a review of memory training interventions and a proposal for a new research study, and preliminary plans for opening an assisted living facility catering to older adults in the LGBTQ community that outlined financial considerations, staff training goals, and patient care plans. Completing the environmental scan project gave students an opportunity to investigate the current state of the career field they are planning to enter, and provided them with a product that they can build upon as they complete the graduate program and begin their careers.

